# A novel diagnostic model for HIV–HTN comorbidity: genomic discovery, clinical validation, and mechanistic elucidation

**DOI:** 10.3389/fmed.2026.1781646

**Published:** 2026-04-09

**Authors:** Aiping Yu, Fangfang Yu, Jida Wang, Jing Song, Yue Hu, Ziyu Wang, Lei Li, Lina Fan, Li Wang, Ping Ma

**Affiliations:** 1 Department of Infectious and Immunology Disease, The Second People’s Hospital Affiliated to Tianjin Medical University, Tianjin, China; 2 First Teaching Hospital of Tianjin University of Traditional Chinese Medicine, Tianjin, China; 3 National Clinical Research Center for Chinese Medicine, Tianjin, China; 4 Tianjin Institute of Hepatology, Tianjin Second People’s Hospital, Tianjin, China; 5 Tianjin University, Tianjin, China; 6 Department of Pharmacy, Tianjin Second People’s Hospital, Tianjin, China

**Keywords:** biomarkers, HIV–HTN comorbidity, immune cell infiltration, machine learning, transcriptomics

## Abstract

**Background:**

Hypertension (HTN) is a frequent comorbidity in people living with Human Immunodeficiency Virus (HIV), yet the shared molecular determinants remain poorly defined. This study aimed to identify diagnostic biomarkers and regulatory networks underlying HIV-HTN comorbidity.

**Methods:**

Transcriptomic datasets from HIV (GSE140713) and HTN (GSE236442) cohorts were analyzed to screen differentially expressed genes. Functional enrichment, GSEA, and PPI analyses were performed to uncover shared pathways and hub genes. Immune cell infiltration was evaluated using CIBERSORT. Transcriptional and post-transcriptional regulatory networks were constructed through NetworkAnalyst, miRNet, and starBase. Key genes were validated in peripheral blood samples from healthy individuals, HIV patients, and HIV-HTN patients using qRT-PCR.

**Results:**

A total of 109 overlapping genes were identified, converging on cytokine-cytokine receptor interaction, IL-17, and NF-κB pathways. Six hub genes (*FOS, PTGS2, TRMT2A, E2F1, FASN, STAB1*) were shared across both diseases. Immune deconvolution showed prominent involvement of DC, monocytes, macrophages, NK/NKT cells, and T-cell subsets. qRT-PCR confirmed consistent upregulation of *TRMT2A, E2F1, FASN*, and *STAB1*, and downregulation of *FOS* and *PTGS2*. A putative ceRNA network was constructed, highlighting several candidate regulatory miRNAs and lncRNAs.

**Conclusion:**

This integrative analysis suggests potential molecular signatures and immune-related regulatory axes that may contribute to HIV-HTN comorbidity, providing hypothesis-generating leads for biomarker discovery and mechanistic follow-up studies.

## Graphical Abstract

**Figure:**
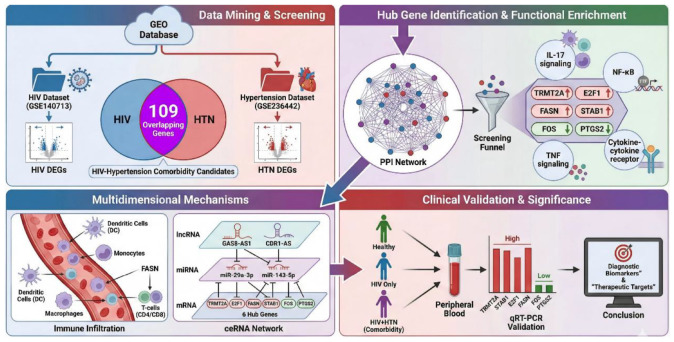
Integrated multi-omics and experimental validation workflow for identifying shared biomarkers and regulatory mechanisms underlying HIV-hypertension (HIV-HTN) comorbidity. Public transcriptomic datasets from HIV (GSE140713) and hypertension (GSE236442) were mined to identify overlapping differentially expressed genes. Protein-protein interaction network analysis and functional enrichment revealed six hub genes (*FOS, PTGS2, TRMT2A, E2F1, FASN*, and *STAB1*) and implicated cytokine-cytokine receptor interaction, IL-17, TNF, and NF-κB signaling pathways. Immune infiltration analysis highlighted the involvement of dendritic cells, monocytes, macrophages, and T-cell subsets. A putative ceRNA regulatory network was further constructed to explore upstream post-transcriptional regulation. Finally, qRT-PCR validation in peripheral blood samples from healthy controls, HIV patients, and HIV-HTN patients confirmed the expression patterns of the identified hub genes, supporting their potential value as diagnostic biomarkers and therapeutic targets in HIV-HTN comorbidity.

## Introduction

Human immunodeficiency virus (HIV) infection remains a major global health burden; UNAIDS estimated that approximately 39 million people were living with HIV by the end of 2023 (1). The advent and widespread implementation of combination antiretroviral therapy (ART) have substantially prolonged survival, transforming HIV from a fatal infectious disease into a chronic condition (2). Consequently, the clinical focus has shifted from opportunistic infections to long-term non-communicable comorbidities (3, 4). Among these, HTN is one of the most prevalent and clinically consequential cardiovascular complications in people living with HIV. Recent meta-analyses report a global HTN prevalence of 35–45% in this population, markedly higher than that in the general population (5). Importantly, HIV–HTN comorbidity accelerates target-organ damage (e.g., renal dysfunction and cardiovascular remodeling) and significantly increases premature morbidity and all-cause mortality (6).

The mechanisms linking HIV infection to HTN are complex and multifactorial (7). Persistent immune activation, chronic low-grade inflammation, endothelial dysfunction, ART-related metabolic disturbances, and host genetic susceptibility have all been implicated (8). Despite increasing recognition of this comorbidity, early detection and refined risk stratification remain challenging. Current approaches largely rely on blood pressure monitoring and routine metabolic profiling, which lack adequate sensitivity and specificity to identify individuals at heightened risk for HIV–HTN comorbidity. Therefore, there is an urgent need for robust molecular biomarkers and predictive models to facilitate early identification, improve patient stratification, and guide targeted interventions (9).

Advances in high-throughput transcriptomic technologies, including next-generation RNA sequencing and gene expression microarrays, enable systematic interrogation of disease-associated molecular signatures (10). Public repositories such as the Gene Expression Omnibus (GEO) provide valuable resources for comparative transcriptomic analyses between patients with comorbid conditions and healthy controls (11). Accumulating evidence indicates that integrative bioinformatics and machine learning frameworks can effectively identify key regulatory genes, develop predictive models, and map pathogenic networks in complex chronic diseases (12, 13). However, the molecular determinants and transcriptional architecture underlying HIV–HTN comorbidity remain insufficiently characterized (14–16).

In the present study, we integrated bioinformatics and machine learning approaches to identify diagnostic candidates and mechanistic genes associated with HIV–HTN comorbidity. Differentially expressed genes (DEGs) were first screened from GEO-based peripheral blood transcriptomic profiles, and candidate biomarkers were subsequently validated in independent cohorts. We further assessed the diagnostic specificity of these biomarkers by comparing HIV–HTN comorbidity with HIV-only and HTN-only groups. Together, these findings provide preliminary molecular evidence relevant to HIV–HTN comorbidity and support further validation of candidate biomarkers in larger, well-characterized cohorts.

## Materials and methods

### Microarray data

The transcriptomic datasets used in this study were downloaded from the Gene Expression Omnibus (GEO) prior to September 2025, including GSE140713 and GSE236442. GSE140713 is an HIV-related dataset that contains mRNA expression profiles from peripheral blood mononuclear cells (PBMCs) obtained from 50 HIV-infected individuals and 7 uninfected healthy controls; according to the original dataset description, HIV-infected participants were receiving antiretroviral therapy (ART) at the time of sampling. GSE236442 is a hypertension (HTN)-related dataset comprising transcriptomic profiles from hypertensive subjects and normotensive controls; participants in this HTN dataset were not receiving antihypertensive pharmacological treatment at sampling (as reported in the original dataset metadata).

Data retrieval and probe annotation. Raw/processed expression matrices and corresponding platform annotation files were retrieved using the GEOquery package in R (v3.5.3). Probe IDs were mapped to official gene symbols based on the corresponding GPL annotation tables provided by GEO. Probes without valid gene symbols were removed. When multiple probes mapped to the same gene symbol, probe-level expression values were collapsed into a single gene-level expression value by taking the average expression using the avereps function in the limma package. If expression values were not log_2_-transformed, log_2_ transformation was applied prior to downstream analyses.

Normalization and batch-effect correction. To reduce technical variability within each dataset, quantile normalization was performed using normalizeBetweenArrays in limma. Batch information (e.g., array/run/plate or other technical identifiers) was extracted from the GEO series metadata when available and used as the batch variable. Where batch effects were present or technical batches were identifiable, batch-effect correction was performed using the ComBat function in the sva package, with the biological group variable (case/control status) retained in the model matrix to avoid removing genuine biological signals. All preprocessing steps were conducted separately within each dataset prior to downstream analyses, and no direct cross-dataset expression-level merging was performed; integrative conclusions were derived from concordant signals identified independently in each dataset.

### Identification of DEGs

Differential expression analysis was performed using the limma package in R. *P*-values were adjusted using the Benjamini–Hochberg false discovery rate (FDR) method. Genes with | log_2_ fold change (log_2_FC)| > 1 and FDR < 0.05 were considered differentially expressed.

### Weighted gene co-expression network analysis

Weighted gene co-expression network analysis (WGCNA) was conducted in the HIV dataset (GSE140713) using the WGCNA package in R to identify co-expression modules associated with HIV status. After preprocessing, genes with low variability were filtered out and the top variable genes were retained for network construction. The soft-thresholding power (β) was selected using the pickSoftThreshold function based on the scale-free topology criterion, with *R*^2^ ≥ 0.85 as the threshold for an approximate scale-free network. An adjacency matrix was constructed and transformed into a topological overlap matrix (TOM). Gene modules were identified by hierarchical clustering with dynamic tree cutting using the following parameters: minModuleSize = 30 and deepSplit = 2. Similar modules were merged using a module eigengene–based clustering approach with mergeCutHeight = 0.25. The significantly associated modules were subsequently intersected with differentially expressed genes (DEGs) to derive robust candidate genes for downstream analyses.

### Functional enrichment analyses

Functional enrichment was carried out in R using the *clusterProfiler* package, encompassing both Gene Ontology (GO) annotation and Kyoto Encyclopedia of Genes and Genomes (KEGG) pathway mapping to distinguish molecular features between the high- and low-risk groups. Terms or pathways with a significance threshold of *P* < 0.05 were retained. In addition, Disease Ontology (DO) enrichment was evaluated through the combined use of *clusterProfiler* and *DOSE*, thereby extending the interpretability of the transcriptomic signatures.

### PPI network construction of overlapping genes

The intersecting genes were imported into the STRING database^1^ to construct a protein–protein interaction (PPI) network. The interaction network was then visualized using Cytoscape software, and key hub genes were identified through topological analysis based on degree centrality and related algorithms.

### CIBERSORT analysis

The CIBERSORT deconvolution framework^2^ was employed to infer the immune cell composition of heterogeneous tissue samples by leveraging predefined transcriptomic signatures. Through this computational strategy, the relative abundances of 22 immune cell subsets were delineated, and their correlations with the transcriptional levels of pivotal genes were systematically assessed in both HIV-infected and hypertensive cohorts. This analysis was designed to illuminate the immune network dynamics and intercellular crosstalk underlying HIV–HTN comorbidity.

### Transcriptional regulatory network analysis of key genes

Potential transcriptional regulators of the hub genes were predicted using the NetworkAnalyst platform,^3^ an integrative web-based tool for comprehensive gene expression analysis and network construction. Hub genes identified from the PPI network were uploaded, and transcription factor (TF)–gene interactions were retrieved based on the JASPAR database embedded within NetworkAnalyst. The resulting regulatory networks were visualized and analyzed to identify key transcription factors potentially governing the expression of hub genes.

### ceRNA regulatory network analysis of core genes

The competing endogenous RNA (ceRNA) network of hub genes was constructed using starBase^4^ and miRNet^5^ databases. First, miRNA–mRNA interactions were predicted for the hub genes, followed by the identification of lncRNA–miRNA interactions. Subsequently, the ceRNA regulatory network was visualized with Cytoscape software to reveal potential lncRNA–miRNA–mRNA interactions.

### Clinical specimens

Blood samples were collected from healthy individuals (*n* = 35; 11 females and 24 males), patients with HIV infection (*n* = 30; all males), and patients with HIV–HTN comorbidity (*n* = 60; 15 females and 45 males), in accordance with the diagnostic criteria of the Chinese Society of Infectious Diseases and the Chinese Guidelines for the Prevention and Treatment of Hypertension. Hypertension was defined as systolic blood pressure ≥ 140 mmHg and/or diastolic blood pressure ≥ 90 mmHg on repeated measurements (Table 1). HIV infection was confirmed by laboratory-based diagnostic gold-standard testing, following a screening-plus-confirmatory algorithm (e.g., HIV-1/2 Ag/Ab screening assay with confirmatory testing and, when indicated, nucleic acid testing). All HIV patients were confirmed to be in the chronic infection stage and were receiving antiretroviral therapy (ART) at the time of sampling. Healthy controls underwent the same HIV testing and blood pressure assessments and were confirmed to be HIV-negative, normotensive, and free of chronic or infectious diseases. Written informed consent was obtained from all participants prior to sample collection. The study protocol was reviewed and approved by the Ethics Committee of Tianjin Second People’s Hospital.

**TABLE 1:** Baseline characteristics.

Characteristic	Healthy controls (*n* = 35)	HIV (*n* = 30)	HIV–HTN (*n* = 60)
Age (years), mean ± SD	36.85 ± 9.82	40.20 ± 8.60	48.15 ± 12.00
Sex, (female/male)	11/24	30/0	15/45
HIV infection duration (years), mean ± SD	/	7.96 ± 4.98	8.93 ± 5.33
HIV clinical stage (definition)	/	Chronic stage	Chronic stage
CD4^+^ T-cell count (cells/μL), mean ± SD		625.28 ± 393.30	580.88 ± 269.11
ART status at sampling	/	Ongoing ART treatment	Ongoing ART treatment
SBP (mmHg), mean ± SD	119.26 ± 12.15	121.07 ± 10.01	157.15 ± 20.77
DBP (mmHg), mean ± SD	77.76 ± 8.37	78.63 ± 5.49	95.08 ± 11.69

Chronic stage: defined according to WHO/CDC criteria; no evidence of acute HIV infection at sampling.

### Real-time polymerase chain reaction assay

Total RNA was isolated from whole blood using TRIzol reagent (Invitrogen) following the manufacturer’s guidelines. The obtained RNA was reverse-transcribed into cDNA with random primers and a commercial kit (Takara, China). Reverse transcription was conducted at 37°C for 15 min and terminated at 85°C for 5 s. Quantitative real-time PCR (qRT-PCR) was subsequently performed with the Power SYBR Green kit (Takara, China) on an ABI 7,500 platform. Gene expression levels were determined by the 2^–Δ^ ^Δ^ Ct approach, with GAPDH serving as the reference gene for normalization. Primer sequences for *TRMT2A*, *PTGS2*, *FOS*, *STAB1*, *FASN*, *E2F1*, and *GAPDH* are listed in Table 2.

**TABLE 2:** Primer sequences used for qRT-PCR.

Gene	Sequence (5’–3’)
*TRMT2A*	Forward: TGGAGTGACCTGCCTCTACTTC
	Reverse: GAAGAAGGCGTGTGGAGAGATC
*PTGS2*	Forward: CGGTGAAACTCTGGCTAGACAG
	Reverse: GCAAACCGTAGATGCTCAGGGA
*FOS*	Forward: GCCTCTCTTACTACCACTCACC
	Reverse: AGATGGCAGTGACCGTGGGAAT
*STAB1*	Forward: GAACCATGTGCCACTGGAAGGC
	Reverse: AGCGGAATCTCCTGGTGCAGTT
*FASN*	Forward: TTCTACGGCTCCACGCTCTTCC
	Reverse: GAAGAGTCTTCGTCAGCCAGGA
*E2F1*	Forward: GGAAACACAAGAGAAGTCCTGCA
	Reverse: CAGCAGTCTCTGCTACTTTCCG
*GAPDH*	Forward: GTCTCCTCTGACTTCAACAGCG
	Reverse: ACCACCCTGTTGCTGTAGCCAA

### Statistical analysis

Statistical analyses were performed using R software (version 3.5.3) and GraphPad Prism (GraphPad Software, United States). Data distribution was assessed using the Shapiro–Wilk normality test. For comparisons among the three clinical groups (healthy controls, HIV, and HIV + HTN), one-way analysis of variance (ANOVA) was applied for normally distributed data, followed by Tukey’s *post-hoc* test. For non-normally distributed data, the Kruskal–Wallis test was used, followed by Dunn’s multiple-comparison test. To account for multiple testing across genes, *P*-values were adjusted using the Benjamini–Hochberg false discovery rate (FDR) method. An adjusted *P*-value (FDR) < 0.05 (or two-sided *P* < 0.05 where applicable) was considered statistically significant.

## Results

### Screening of differentially expressed genes in HIV and HTN

**FIGURE 1:**
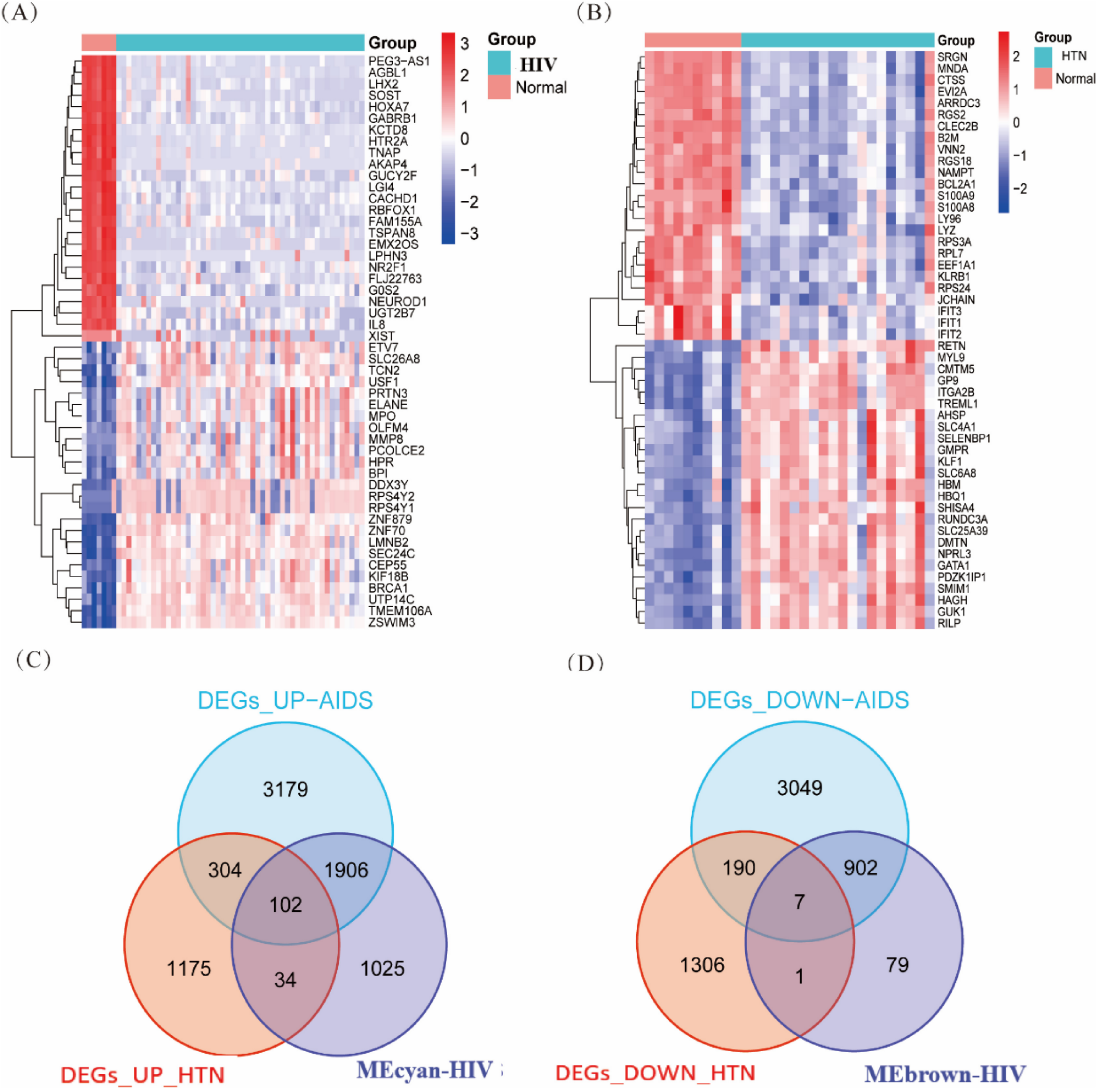
Differential expression and overlap analysis in HIV and hypertension datasets. **(A)** DEG heatmap for HIV vs. healthy controls (GSE140713). **(B)** DEG heatmap for HTN vs. normotensive controls (GSE236442). **(C)** Venn overlap of upregulated DEGs across HIV, HTN, and the MEcyan–HIV module. **(D)** Venn overlap of downregulated DEGs across HIV, HTN, and the MEbrown–HIV module.

In order to identify potential biomarkers and unravel the molecular mechanisms linking HIV and HTN, we conducted a comprehensive analysis of transcriptomic datasets from both conditions. The aim was to screen for differentially expressed genes (DEGs) that could provide insights into shared biological pathways. The HIV dataset (GSE140713) included peripheral blood samples from 50 HIV-infected individuals and 7 uninfected controls. After preprocessing (normalization and batch effect correction), differential expression analysis was performed using the Limma package in R, with thresholds set at | log_2_FC| > 1 and FDR < 0.05. This analysis identified 8,739 DEGs: 4,591 upregulated and 4,148 downregulated. Functional enrichment analysis indicated significant involvement of these genes in immune regulation and apoptosis, suggesting their role in HIV pathogenesis (Figure 1A).

Similarly, the HTN dataset (GSE236442) was processed using the same pipeline, revealing 3,119 DEGs, with 1,615 upregulated and 1,504 downregulated genes. The distribution patterns highlighted substantial overlap in inflammatory and cardiovascular pathways, pointing to extensive transcriptomic changes in HTN. These findings provide candidate genes for investigating the shared molecular mechanisms between HIV and HTN (Figure 1B).

### Convergence of gene expression in HIV and HTN

Comparing the upregulated genes in both datasets, we identified 102 transcripts consistently elevated in both HIV and HTN (Figure 1C), suggesting convergent molecular alterations related to immune activation and inflammation. Likewise, 7 downregulated genes were shared between the two conditions (Figure 1D), indicating potential impairments in shared regulatory pathways. These overlapping gene signatures highlight common transcriptomic disturbances that may underlie the comorbidity of HIV and HTN.

### Functional enrichment and PPI network analyses of intersecting genes

**FIGURE 2:**
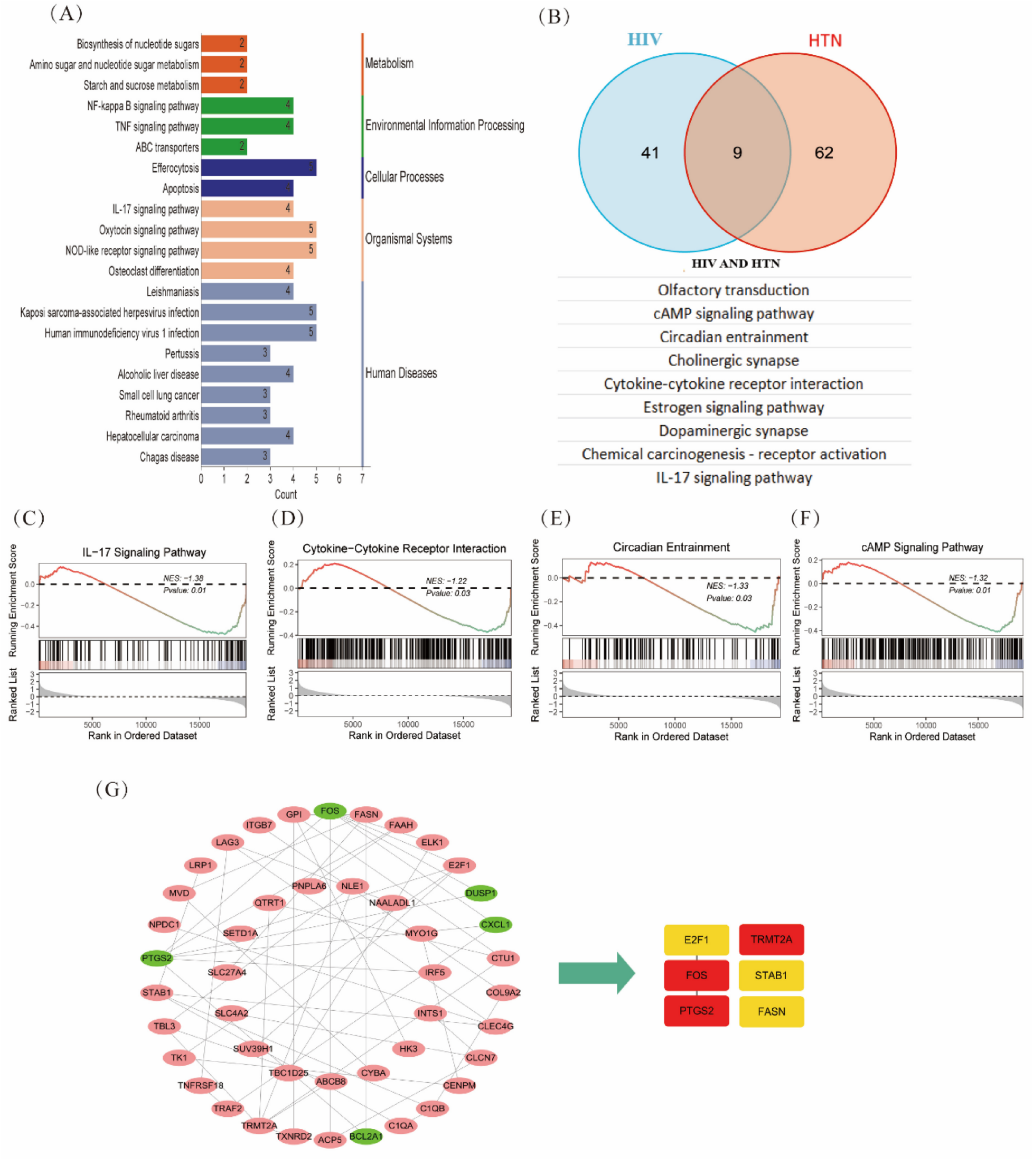
Integrated bioinformatics analysis of HIV and HTN. **(A)** KEGG enrichment of 109 DEGs (ClusterProfiler, R). **(B)** Venn overlap of enriched pathways between the HIV and HTN datasets. **(C–F)** GSEA of shared pathways (IL-17 signaling, cytokine–cytokine receptor interaction, circadian entrainment, and cAMP signaling). **(G)** PPI network and hub-gene identification (STRING; MCC algorithm).

Functional enrichment of the 109 overlapping DEGs was conducted to identify key pathways involved in both diseases. KEGG analysis revealed significant enrichment in multiple pathways, including NF-kappa B, TNF, ABC transporters, IL-17, oxytocin, and NOD-like receptor signaling, as well as cellular processes such as efferocytosis and apoptosis. These findings suggest shared biological processes contributing to the pathogenesis of both HIV and HTN, with links to human diseases like HIV-1 infection and KSHV infection (Figure 2A).

Further GSEA revealed 50 enriched pathways in the HIV group and 71 in the HTN group. Nine pathways were common to both, including olfactory transduction, cAMP signaling, circadian entrainment, cholinergic synapse, cytokine–cytokine receptor interaction, estrogen signaling, dopaminergic synapse, chemical carcinogenesis (receptor activation), and IL-17 signaling (Figures 2B–F). These common pathways indicate overlapping molecular mechanisms in immune and inflammatory responses in both conditions.

Finally, PPI network analysis of the DEGs revealed the top six hub genes—FOS, *PTGS2*, *TRMT2A*, *E2F1*, *FASN*, and *STAB1*—that were involved in both HIV and HTN (Figure 2G).

### Association between genes and immune cell infiltration

In the context of HIV infection, the expression of *FOS*, *PTGS2*, *TRMT2A*, *E2F1*, *FASN*, and *STAB1* shows significant associations with various immune cell subsets. Specifically, *FOS* expression was significantly downregulated in CD4^+^ naïve, CD4^+^ T, CD8^+^ naïve, CD8^+^ T cells, DCs, iTregs, NK cells, NKT cells, nTregs, follicular helper Tfh cells, and Tr1 cells, while it was upregulated in macrophages, monocytes, and neutrophils (Supplementary Figures S1A–N). Similarly, *PTGS2* was downregulated in CD4 ^+^ naïve, CD4 ^+^ T, CD8 ^+^ naïve, CD8 ^+^ T, iTregs, NK cells, NKT cells, nTregs, Tfh cells, Th2 cells, and Tr1 cells, but upregulated in macrophages, monocytes, and neutrophils (Supplementary Figures 2A–N). *TRMT2A* exhibited downregulation in CD8 ^+^ naïve, DCs, monocytes, neutrophils, and nTregs, but upregulation in CD4 ^+^ T, CD8 ^+^ T, iTregs, Tfh, Th2, and Tr1 cells (Supplementary Figure 3A–K). *E2F1* was predominantly downregulated in B cells and CD4^+^ naïve T cells (Supplementary Figures 4A,B). *FASN* was downregulated in macrophages, monocytes, and neutrophils but upregulated in CD4^+^ naïve, CD4^+^ T, CD8^+^ T, iTregs, Tfh, Th2, and Tr1 cells (Supplementary Figures 4C–L). *STAB1* expression was reduced in CD8 ^+^ naïve and macrophages (Supplementary Figures 4M,N).

In the context of HTN and HIV, *FOS*, *PTGS2*, *TRMT2A*, *E2F1*, *FASN*, and *STAB1* show significant associations with various immune cell subsets. Specifically, *FOS* expression was significantly downregulated in DCs but upregulated in monocytes (Supplementary Figures 5A,B). *PTGS2* was downregulated in B cells, DCs, and monocytes, but upregulated in CD8 ^+^ T cells and neutrophils (Supplementary Figures 5C–G). *TRMT2A* was downregulated in monocytes, neutrophils, and Th17 cells, with upregulation in DCs (Supplementary Figures 5H–K). *E2F1* expression was downregulated in DCs, while it was upregulated in neutrophils (Supplementary Figures 5L,M). *FASN* was downregulated in monocytes and neutrophils, but upregulated in CD8^+^ naïve, CD8^+^ T cells, and DCs (Supplementary Figures 5N–R). *STAB1* expression was notably upregulated in DCs (Supplementary Figure 5S).

**FIGURE 3:**
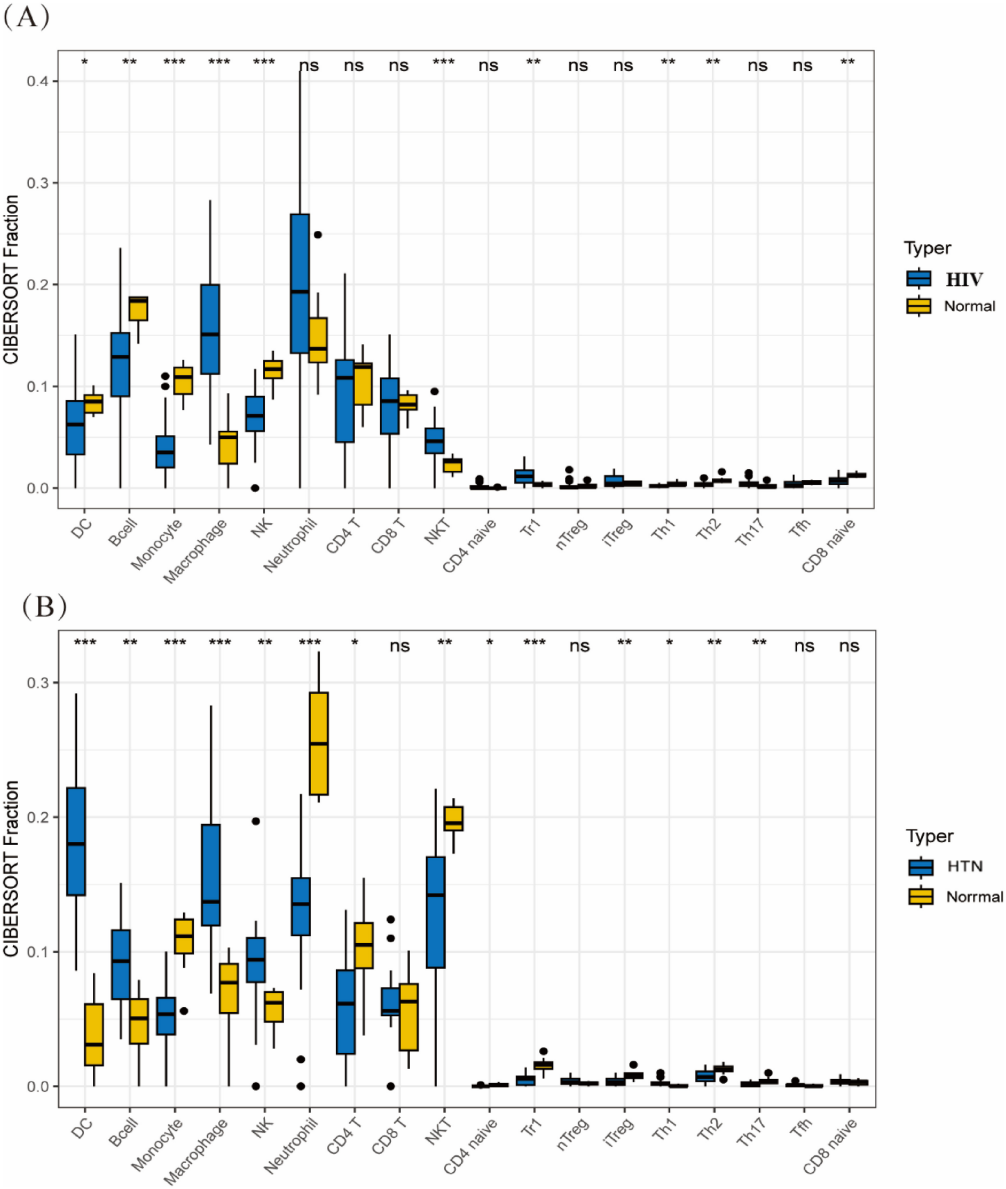
Immune cell infiltration analysis in HIV and HTN. **(A,B)** CIBERSORT-based immune infiltration analysis comparing immune cell proportions between **(A)** HIV and controls and **(B)** HTN and controls (*P < 0.05, **P < 0.01, ***P < 0.001, ****P < 0.0001).

Comprehensive immune profiling revealed that the progression of both HTN and HIV is intricately regulated by various immune cell subsets, including DCs, B cells, macrophages, monocytes, NK cells, NKT cells, iTregs, Th1 cells, and Th2 cells (Figures 3A,B).

### Validation of the expression of six diagnostic genes

**FIGURE 4:**
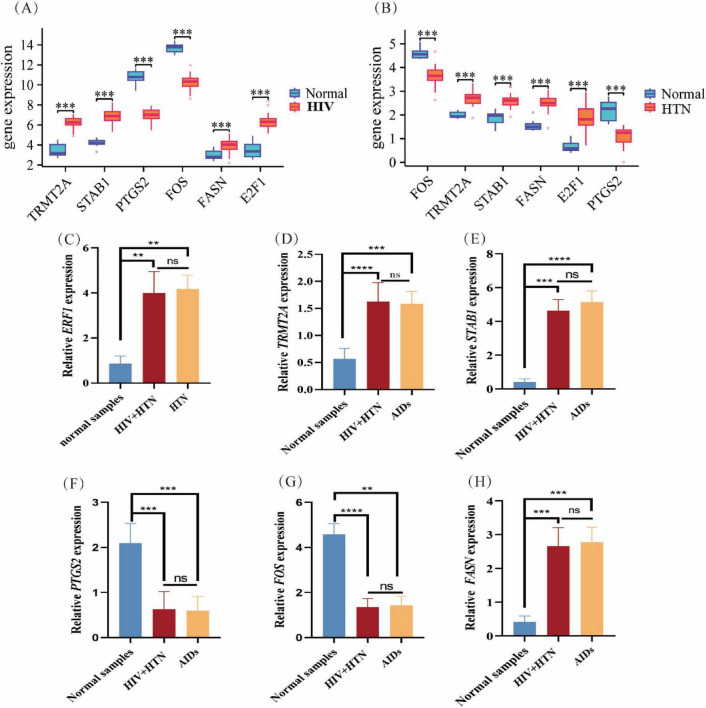
Validation of key diagnostic gene expression in HIV and HTN. **(A,B)** Differential expression analysis of six hub genes (*FOS*, *PTGS2*, *TRMT2A*, *E2F1*, *FASN*, and *STAB1*) between normal and disease groups in the HIV and HTN datasets. **(C–H)** qRT-PCR validation of hub gene expression (*E2F1*, *TRMT2A*, *STAB1*, *PTGS2*, *FOS*, and *FASN*) in peripheral blood samples from healthy individuals, HIV patients, and patients with HIV complicated by HTN (*P < 0.05, **P < 0.01, ***P < 0.001, ****P < 0.0001).

We examined the expression profiles of key diagnostic genes related to HIV and HTN in the dataset. The results showed that *TRMT2A*, *STAB1*, *E2F1*, and *FASN* were upregulated, while *FOS* and *PTGS2* were downregulated in both conditions (Figures 4A,B). These trends were consistently observed in peripheral blood samples from healthy individuals, HIV patients, and those with HIV complicated by HTN, supporting the findings in the dataset (Figures 4C–H).

### Construction of the mRNA–miRNA–lncRNA regulatory network

**FIGURE 5:**
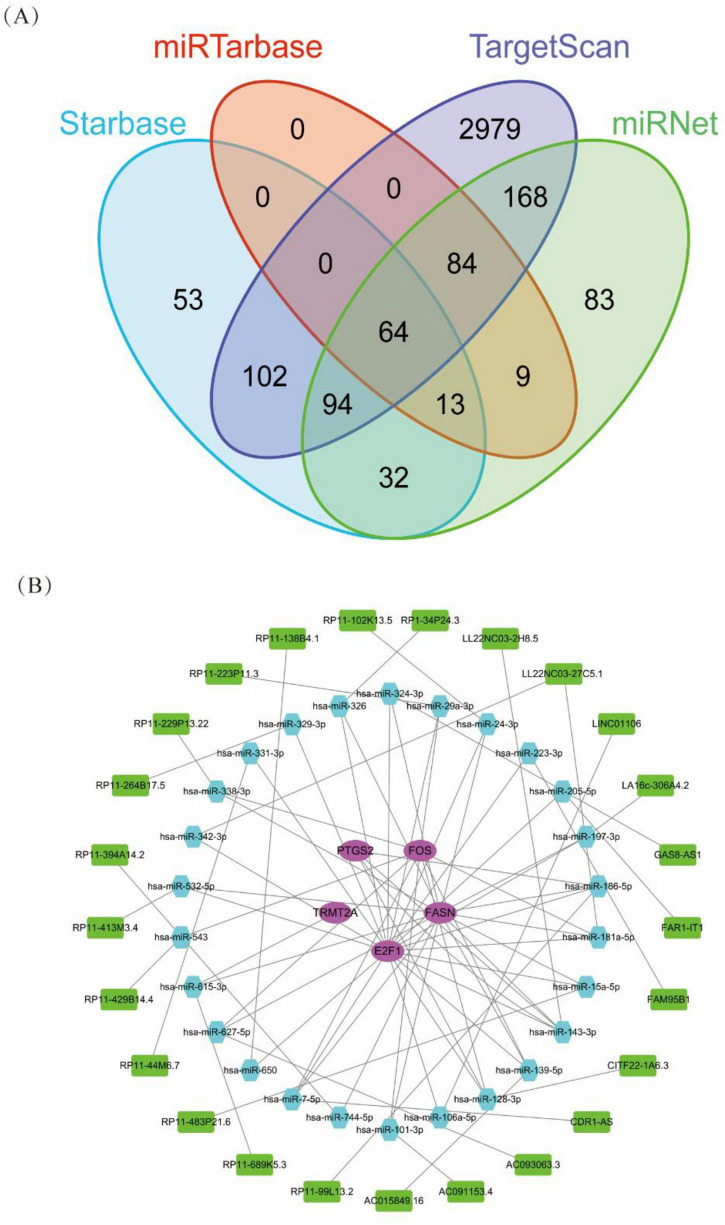
Construction of the lncRNA–miRNA–mRNA regulatory network associated with HIV and HTN. **(A)** Venn overlap of predicted miRNAs targeting key diagnostic genes across miRTarBase, TargetScan, StarBase, and miRNet. **(B)** lncRNA–miRNA–mRNA network linking hub genes with their candidate miRNAs and upstream lncRNAs.

To identify potential upstream regulatory miRNAs targeting the key diagnostic genes, we analyzed four independent databases—miRTarBase, TargetScan, StarBase, and miRNet. The Venn diagram (Figure 5A) shows the overlap among these datasets. A total of 64 miRNAs were identified across all four databases, while 94, 84, 168, and 53 miRNAs were shared between three of the databases, respectively. Notably, miRTarBase yielded no unique targets, whereas TargetScan predicted the largest number of candidates (*n* = 2,979). These overlapping miRNAs were selected as high-confidence candidates for further regulatory network construction and functional validation.

To further explore the post-transcriptional regulatory mechanisms of key diagnostic genes, we constructed a comprehensive mRNA–miRNA–lncRNA regulatory network. This network revealed that the hub genes *FOS*, *PTGS2*, *TRMT2A*, *E2F1*, and *FASN* were targeted by multiple miRNAs, including hsa-miR-29a-3p, hsa-miR-205-5p, hsa-miR-143-5p, hsa-miR-181a-5p, and hsa-miR-342-3p. Additionally, several lncRNAs, such as GAS8-AS1, CDR1-AS, FAR1-IT1, LINC01106, and LL22NC03-27C5.1, were identified as upstream regulators of these miRNAs, suggesting the formation of a ceRNA regulatory axis. These findings highlight the potential role of the lncRNA–miRNA–mRNA network in mediating the shared pathogenic mechanisms of HIV and HTN (Figure 5B).

## Discussion

The present study systematically delineated the molecular landscape and immune regulatory mechanisms underlying the comorbidity of HIV infection and HTN (17, 18). By integrating transcriptomic data, bioinformatic analysis, and clinical validation, we identified six hub genes (*FOS*, *PTGS2*, *TRMT2A*, *E2F1*, *FASN*, and *STAB1*) that may serve as potential diagnostic biomarkers and mechanistic mediators of this comorbidity. Moreover, our construction of a ceRNA regulatory network involving these genes revealed multilayered post-transcriptional interactions mediated by miRNAs and lncRNAs. Collectively, these findings provide new insights into the molecular basis of HIV–HTN comorbidity and highlight the potential value of immune–metabolic cross-talk in its pathogenesis.

Chronic immune activation is a hallmark of HIV infection, persisting even under effective antiretroviral therapy (19–21). This sustained inflammatory milieu—characterized by elevated cytokines such as IL-6, TNF-α, and IFN-γ—promotes endothelial dysfunction, vascular stiffness, and metabolic dysregulation, which are also central features of HTN (22–24). Our CIBERSORT-based immune deconvolution revealed overlapping immune perturbations in both HIV and HTN cohorts, including increased proportions of monocytes, macrophages, and dendritic cells, as well as altered T-cell subset distributions (25–27). These findings are consistent with prior reports demonstrating that activated monocytes and tissue macrophages release pro-inflammatory cytokines that contribute to vascular remodeling and increased peripheral resistance in hypertensive patients (28, 29). Similarly, excessive activation of dendritic cells (DCs) in HIV infection perpetuates antigen presentation and T-cell stimulation, reinforcing systemic inflammation (30). Hence, the convergence of innate and adaptive immune dysregulation likely constitutes the immunological foundation of HIV–HTN comorbidity.

Among the six hub genes identified, *FOS* and *PTGS2* were downregulated, whereas *TRMT2A*, *E2F1*, *FASN*, and *STAB1* were upregulated in both disease conditions. FOS, a core transcription factor of the AP-1 complex, plays a pivotal role in immune activation and vascular damage in both HIV infection and hypertension. In HIV infection, FOS promotes chronic immune activation and inflammation by regulating immune cell proliferation, apoptosis, and cytokine release, reflecting excessive immune activation or exhaustion. Meanwhile, in hypertension, *FOS* contributes to vascular remodeling and atherosclerosis by promoting the proliferation and migration of vascular smooth muscle cells, thereby exacerbating endothelial dysfunction and vascular injury. Consequently, *FOS* serves as a critical link between immune activation and vascular damage in the pathophysiology of both conditions (31). Reduced *FOS* expression in immune cells may reflect exhaustion or impaired anti-inflammatory signaling, consistent with findings in chronic inflammatory states and viral infections (32). Similarly, *PTGS2* (cyclooxygenase-2) plays a crucial role in the synthesis of prostaglandins, which are key mediators of inflammation and vascular homeostasis. In HIV infection, the upregulation of *PTGS2* promotes chronic low-grade inflammation, enhances immune cell activation, and contributes to tissue damage, thereby playing a role in the pathogenesis of HIV-associated chronic diseases. In hypertension, *PTGS2* inhibits prostaglandin-mediated vasodilation, leading to increased oxidative stress and endothelial dysfunction, which further exacerbates vascular injury (33).

Conversely, *TRMT2A* (tRNA methyltransferase 2A) is an enzyme involved in the methylation of tRNA molecules, playing a critical role in maintaining protein translation fidelity and cellular stress responses. In the context of HIV infection, *TRMT2A* is thought to be upregulated as a compensatory mechanism to enhance tRNA stability and translation accuracy under the stress of viral replication (34). Its elevated expression may help cells cope with the increased demand for protein synthesis during viral infection. In hypertension, *TRMT2A* may also be involved in the cellular adaptation to increased oxidative stress and metabolic alterations (35). *E2F1* (E2F transcription factor 1) is a pivotal transcription factor that plays a crucial role in regulating the cell cycle, particularly in the progression from the G1 phase to the S phase. It is also involved in various cellular processes, including DNA repair, apoptosis, and endothelial cell proliferation. In the context of HIV infection, E2F1 is upregulated as part of the immune response and cellular stress induced by the viral replication process. Its elevated expression may promote the proliferation of immune and endothelial cells, contributing to the chronic inflammation and vascular remodeling commonly observed in HIV-infected individuals (36). In hypertension, *E2F1* is associated with endothelial cell proliferation and apoptosis, playing a role in vascular remodeling and endothelial dysfunction. The upregulation of *E2F1* in both HIV and hypertension suggests its involvement in regulating cell cycle progression and contributing to vascular damage in both conditions, linking immune activation, inflammation, and vascular injury (37).

*FASN* is a pivotal enzyme in fatty acid synthesis, playing a central role in lipid metabolism and cellular membrane formation. It has been increasingly recognized as a key regulator of immune responses and metabolic processes. In the context of HIV infection, *FASN* is upregulated in activated immune cells, facilitating lipid biosynthesis to support cell membrane formation and energy production (38). This upregulation may also contribute to the inflammation and metabolic reprogramming observed in HIV-infected individuals. In hypertension, *FASN* is involved in altered lipid metabolism, often linked to the development of metabolic syndrome. Elevated *FASN* expression is associated with pro-inflammatory lipid profiles, which can exacerbate vascular inflammation and contribute to endothelial dysfunction. Finally, *STAB1* (stabilin-1) is a scavenger receptor predominantly expressed on macrophages and endothelial cells, involved in the endocytosis of modified lipids and apoptotic debris. It plays a key role in maintaining tissue homeostasis by clearing cellular debris and modulating immune responses. In the context of HIV infection, *STAB1* is upregulated as part of the inflammatory response, reflecting macrophage activation and dysfunction. This upregulation may contribute to chronic inflammation and tissue damage commonly seen in HIV-infected individuals (39). Similarly, in hypertension, *STAB1* is implicated in the dysregulation of vascular clearance mechanisms, potentially leading to the accumulation of modified lipids and apoptotic cells, which exacerbates vascular inflammation and accelerates endothelial dysfunction. Thus, *STAB1* plays an important role in both the immune response and vascular damage associated with HIV infection and hypertension, acting as a link between immune activation and vascular injury.

Pathway enrichment analyses revealed significant overlap between HIV and HTN in multiple signaling cascades, notably the cytokine–cytokine receptor interaction, IL-17, NF-κB, and TNF pathways. These pathways are central mediators of inflammatory and immune responses (40, 41). The IL-17 axis, predominantly driven by Th17 cells, is particularly relevant, as elevated IL-17 levels contribute to vascular inflammation, endothelial dysfunction, and arterial stiffness. Likewise, TNF and NF-κB signaling cascades amplify the production of adhesion molecules and inflammatory mediators, leading to vascular smooth muscle proliferation and extracellular matrix remodeling (42). Notably, our results also identified enrichment in circadian rhythm and cAMP signaling pathways, underscoring the contribution of neuroendocrine and metabolic dysregulation to the disease process. These shared signaling mechanisms support the concept that HIV–HTN comorbidity represents a syndromic interplay between immune–inflammatory activation and cardiometabolic remodeling.

The integration of transcriptomic and immune infiltration analyses highlights the diagnostic potential of immune-cell–gene interactions (43). Our findings demonstrated that *FOS* and *PTGS2* expression negatively correlated with CD4^+^ and CD8^+^ T-cell subsets, while *FASN* and *E2F1* showed positive associations with dendritic cells and regulatory T cells (Tregs). Such opposing expression patterns reflect immune exhaustion and compensatory regulatory responses. The dynamic balance between pro-inflammatory and regulatory cell subsets likely determines the clinical trajectory of HIV–HTN comorbidity. Therapeutically, interventions aimed at restoring immune homeostasis—such as IL-6 blockade, TNF inhibition, or metabolic modulators targeting FASN—may hold promise for reducing vascular injury in HIV with HTN (44). Furthermore, these immune-associated transcriptional markers could serve as minimally invasive blood-based indicators for early diagnosis and patient stratification.

Beyond transcriptional regulation, our integrative ceRNA analysis suggests a complex post-transcriptional regulatory landscape involving miRNAs and lncRNAs (45). The candidate miRNAs identified— including hsa-miR-29a-3p, hsa-miR-143-5p, and hsa-miR-181a-5p—have been reported to participate in pathways related to endothelial homeostasis, inflammatory signaling, and lipid metabolism. For instance, miR-29a has been linked to extracellular matrix remodeling and vascular fibrosis, whereas the miR-143 and miR-181 families have been associated with macrophage polarization and T-cell differentiation in prior studies (46–48). The lncRNAs highlighted in our network, such as GAS8-AS1 and CDR1-AS, have also been implicated in cardiovascular and immune-related regulation, potentially through miRNA “sponging” effects (49, 50).

Importantly, the lncRNA–miRNA–mRNA network presented here is prediction-based and therefore should be interpreted as hypothesis-generating. Nevertheless, it provides a plausible framework by which non-coding RNAs may fine-tune gene-expression programs relevant to HIV–HTN comorbidity. Collectively, these putative ceRNA interactions may represent one potential regulatory layer linking immune activation with endothelial dysfunction, and they warrant further experimental validation (e.g., expression correlation in independent cohorts, luciferase reporter assays, and loss-/gain-of-function studies) before being considered actionable therapeutic targets.

The validation of *TRMT2A*, *STAB1*, *E2F1*, *FASN*, *FOS*, and *PTGS2* expression in clinical blood samples reinforces their diagnostic and translational potential. The observed concordance between computational prioritization and qRT-PCR validation supports the internal consistency of our integrative framework. Because peripheral blood transcriptomic profiling is relatively accessible, the identified signatures may be further evaluated as inputs for multivariable risk models to aid in stratifying individuals at higher risk of developing hypertension during chronic HIV infection. Importantly, the therapeutic implications remain hypothesis-generating at this stage. While pathway-level findings point to potentially druggable processes (e.g., lipid metabolism, NF-κB–related inflammatory signaling, and non-coding RNA regulation), the clinical relevance and safety of targeting these axes in people living with HIV will require rigorous validation in independent cohorts and dedicated mechanistic and pharmacologic studies before any translational claims can be made.

Despite the strengths of this integrative study, several limitations should be acknowledged. First, the public transcriptomic datasets were derived from peripheral blood rather than vascular or other tissue-specific specimens, which may limit inference regarding localized vascular remodeling and injury. Second, although qRT-PCR validation supported the observed expression patterns, larger, multicenter cohorts are required to establish robust diagnostic thresholds, assess predictive performance, and improve generalizability. Third, the ceRNA network was constructed based on computational inference; experimental validation (e.g., dual-luciferase reporter assays, RNA pull-down, and gain-/loss-of-function studies) is needed to confirm direct interactions and functional relevance. Finally, prospective longitudinal studies integrating metabolic, immunological, and clinical parameters will be essential to clarify temporal dynamics and strengthen causal inference linking immune dysregulation, transcriptional alterations, and the development of hypertension in people living with HIV.

## Conclusion

In summary, this study provides a comprehensive molecular characterization of HIV–HTN comorbidity, identifying key genes and immune networks that underpin their shared pathogenic mechanisms. The hub genes *FOS*, *PTGS2*, *TRMT2A*, *E2F1*, *FASN*, and *STAB1* constitute central nodes linking immune activation, metabolic remodeling, and vascular dysfunction. The integrated ceRNA network further reveals novel post-transcriptional regulatory mechanisms involving miRNAs and lncRNAs. These findings not only advance our understanding of the immunometabolic interplay in HIV–associated HTN but also open new avenues for biomarker development and targeted therapy. Future translational studies focusing on the modulation of these molecular pathways may facilitate precision management of cardiovascular complications in people living with HIV.

## Data Availability

The original contributions presented in the study are included in the article/Supplementary material, further inquiries can be directed to the corresponding authors.

## Footnotes

1
https://string-db.org/

2
http://cibersort.stanford.edu/

3
https://www.networkanalyst.ca/

4
https://starbase.sysu.edu.cn/

5
https://www.mirnet.ca/
