# Supplementary material for: A novel diagnostic model for HIV–HTN comorbidity: genomic discovery, clinical validation, and mechanistic elucidation
Source: Front Med (Lausanne). 2026 Apr 9;13:1781646. doi: 10.3389/fmed.2026.1781646 (PMC13102566; doi:10.3389/fmed.2026.1781646)
Supplement: Supplementary file 1 [file Supplementary_file_1.docx]

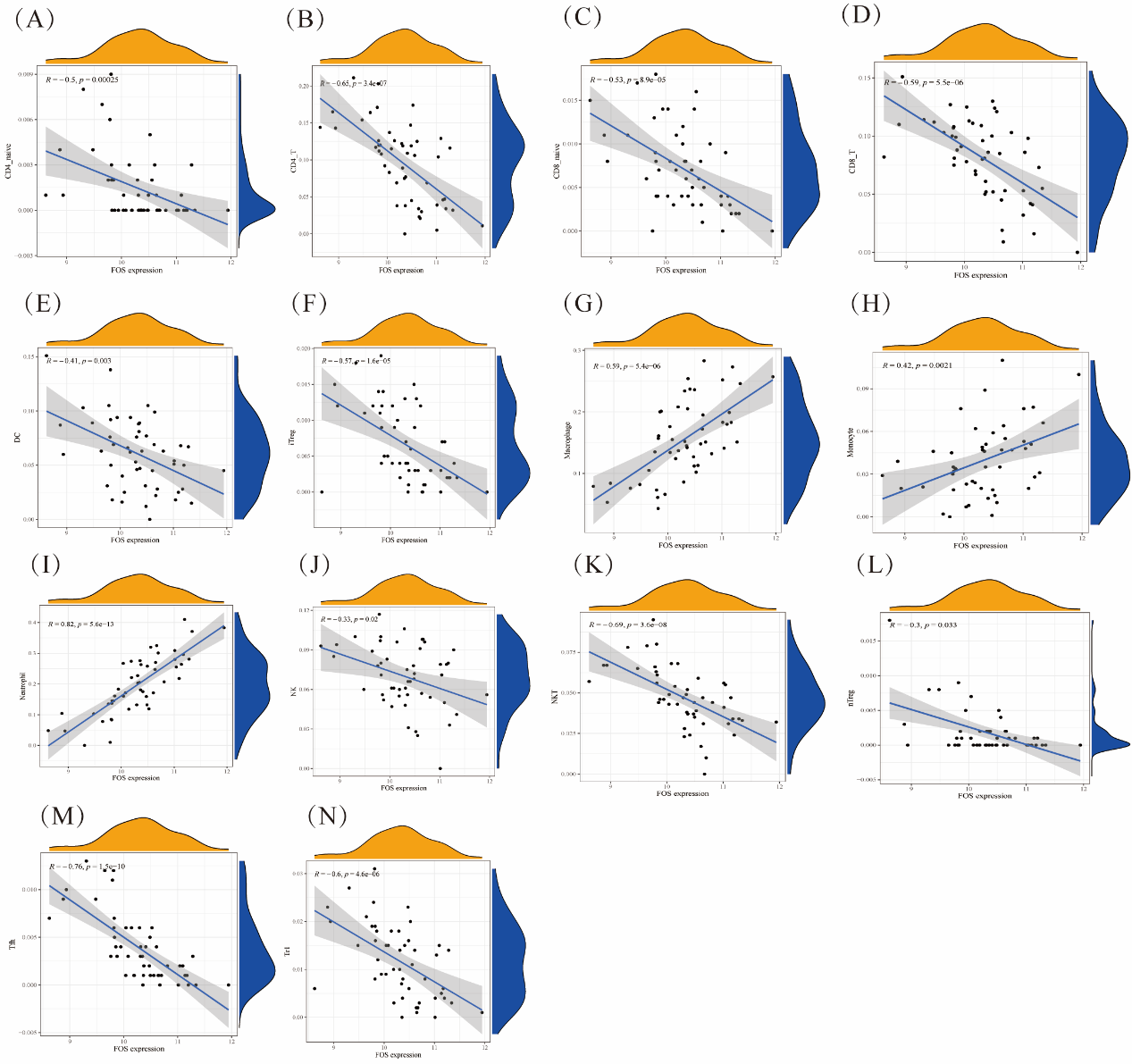


**Supplementary Fig. 1. Correlation analysis between FOS expression and immune cell infiltration in HIV infection.** (A–N) Spearman correlation analysis of FOS expression with multiple immune cell subsets, including CD4⁺ naïve T cells, CD4⁺ T cells, CD8⁺ naïve T cells, CD8⁺ T cells, dendritic cells (DCs), induced regulatory T cells (iTregs), macrophages, monocytes, naïve regulatory T cells (nTregs), follicular helper T cells (Tfh), natural killer (NK) cells, natural killer T (NKT) cells, neutrophils, and type 1 regulatory T cells (Tr1).


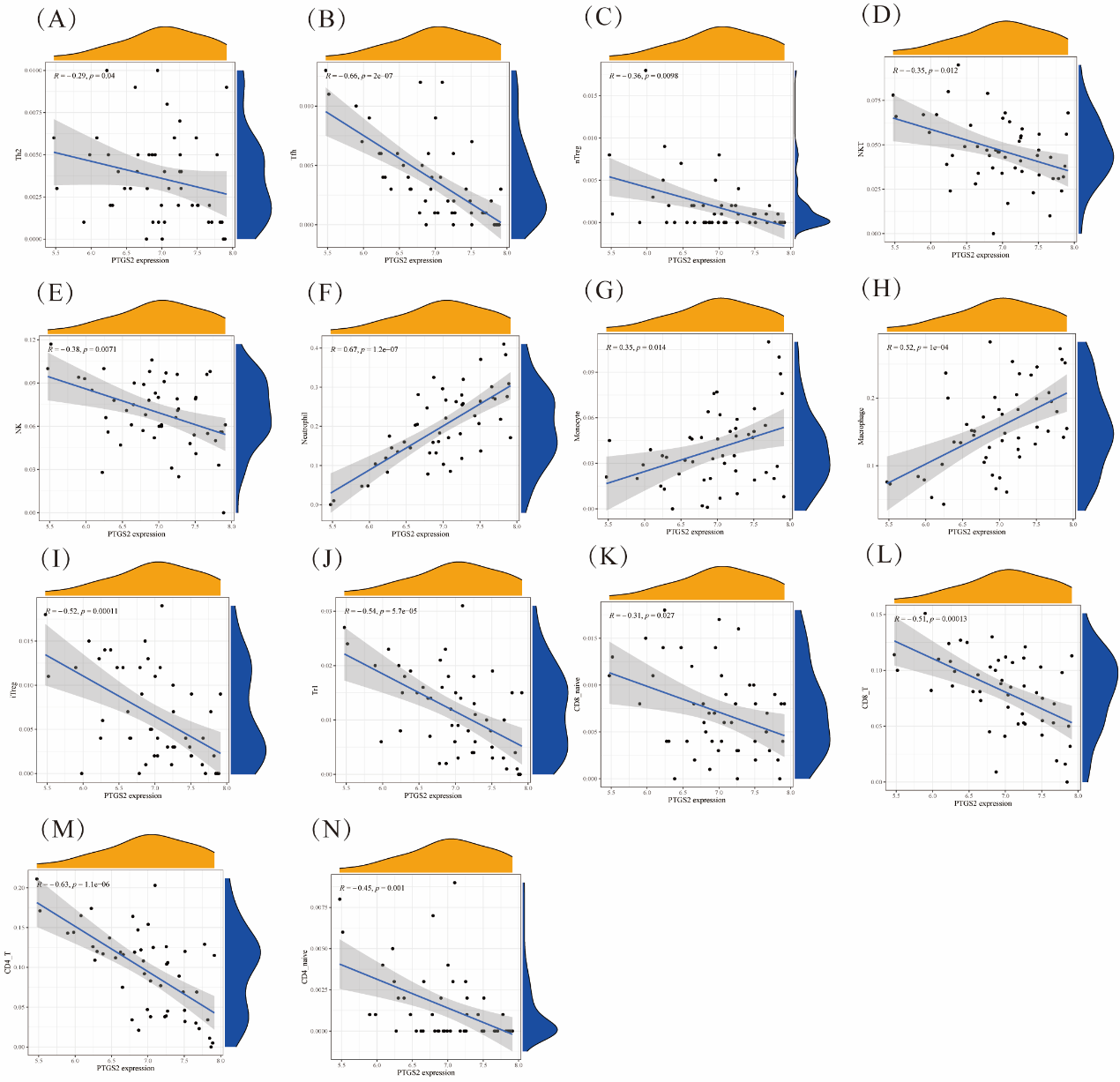


**Supplementary Fig. 2. Correlation analysis between PTGS2 expression and immune cell infiltration in HIV infection.** (A–N) Spearman correlation analysis of PTGS2 expression with multiple immune cell subsets, including CD4⁺ naïve T cells, CD4⁺ T cells, CD8⁺ naïve T cells, CD8⁺ T cells, induced regulatory T cells (iTregs), macrophages, monocytes, naïve regulatory T cells (nTregs), follicular helper T cells (Tfh), natural killer (NK) cells, natural killer T (NKT) cells, neutrophils, T helper type 2 (Th2) cells, and type 1 regulatory T cells (Tr1).


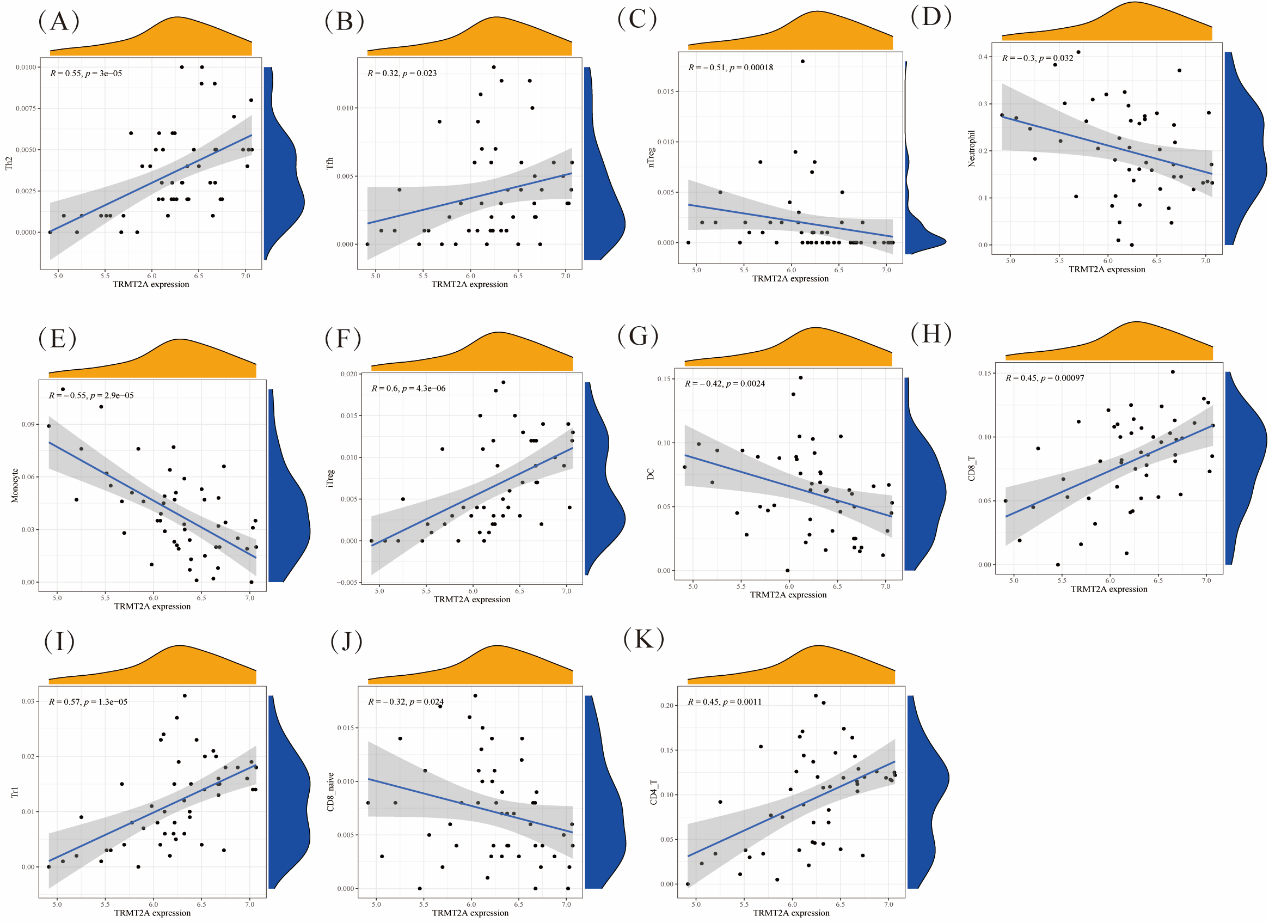


**Supplementary Fig. 3. Correlation analysis between TRMT2A expression and immune cell infiltration in HIV infection.** (A–K) Spearman correlation analysis of TRMT2A expression with multiple immune cell subsets, including CD8⁺ naïve T cells, dendritic cells (DCs), monocytes, neutrophils, naïve regulatory T cells (nTregs), CD4⁺ T cells, CD8⁺ T cells, induced regulatory T cells (iTregs), follicular helper T cells (Tfh), T helper type 2 (Th2) cells, and type 1 regulatory T cells (Tr1).


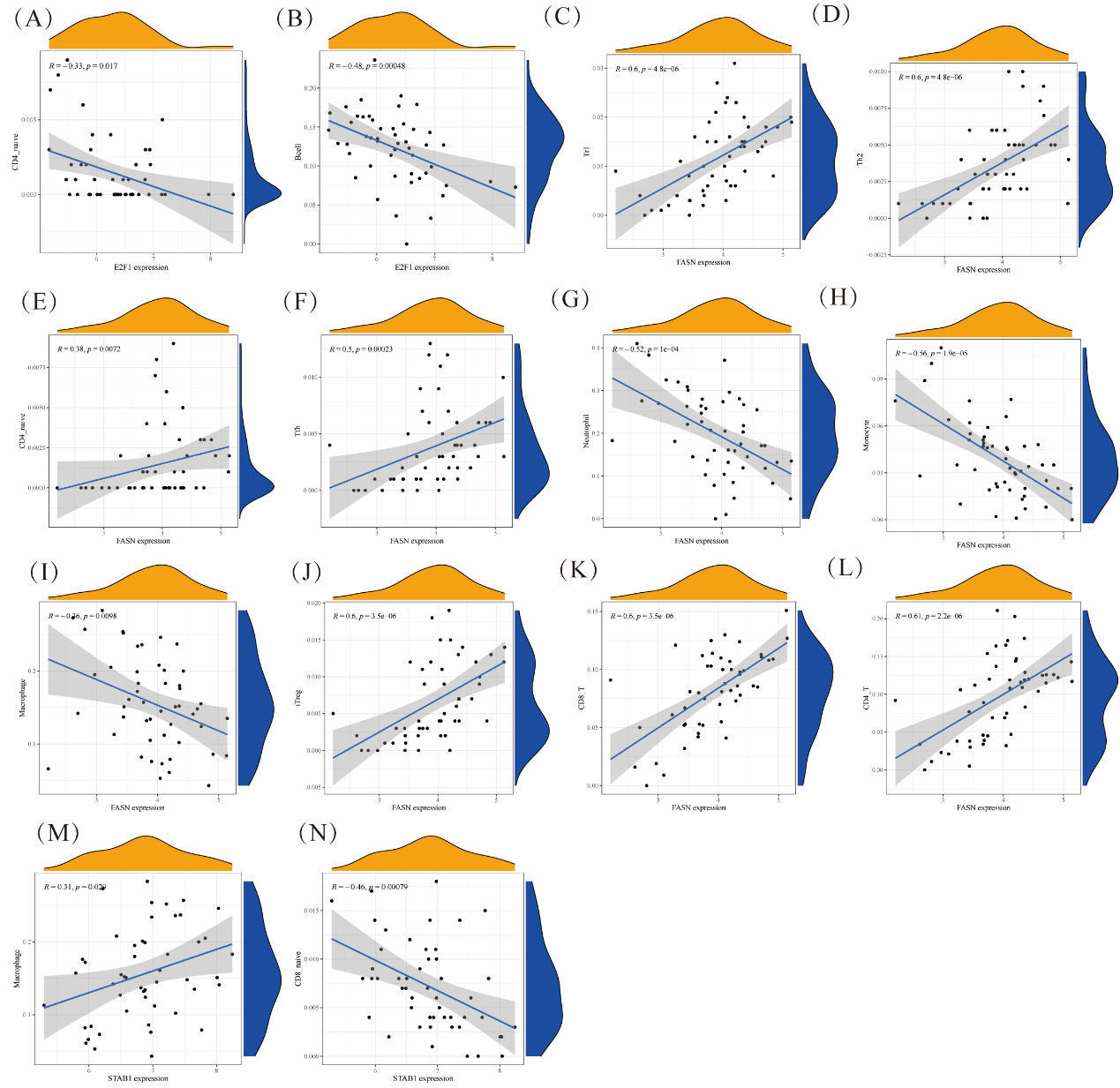


**Supplementary Fig. 4. Correlation analysis between E2F1, FASN, and STAB1 expression and immune cell infiltration in HIV infection.** (A–B) Spearman correlation analysis of E2F1 expression with B cells and CD4⁺ naïve T cells. (C–L) Spearman correlation analysis of FASN expression with multiple immune cell subsets, including macrophages, monocytes, neutrophils, CD4⁺ naïve T cells, CD4⁺ T cells, CD8⁺ T cells, induced regulatory T cells (iTregs), follicular helper T cells (Tfh), T helper type 2 (Th2) cells, and type 1 regulatory T cells (Tr1). (M–N) Spearman correlation analysis of STAB1 expression with CD8⁺ naïve T cells and macrophages.


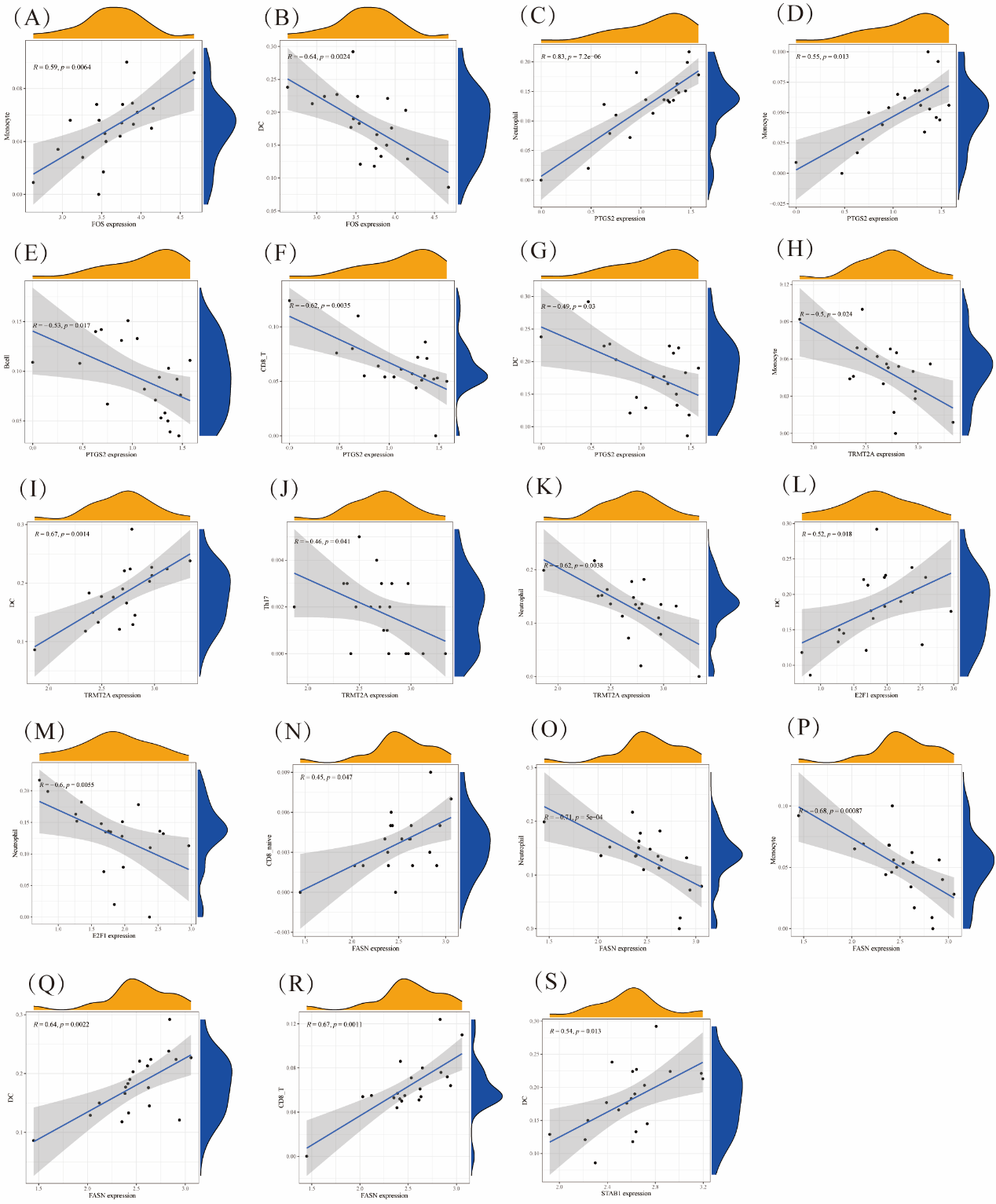


**Supplementary Fig. 5. Correlation analysis between hub gene expression and immune cell infiltration in HIV–hypertension comorbidity.** (A–S) Spearman correlation analysis of FOS, PTGS2, TRMT2A, E2F1, FASN, and STAB1 expression with various immune cell subsets, including B cells, dendritic cells (DCs), monocytes, neutrophils, CD8⁺ T cells, CD8⁺ naïve T cells, and T helper 17 (Th17) cells.
